# Heatwaves: does global research reflect the growing threat in the light of climate change?

**DOI:** 10.1186/s12992-023-00955-4

**Published:** 2023-08-11

**Authors:** Doris Klingelhöfer, Markus Braun, Dörthe Brüggmann, David A. Groneberg

**Affiliations:** Institute of Occupational, Social and Environmental Medicine, Goethe University Frankfurt, Theodor-Stern-Kai 7, 60590 Frankfurt, Germany

**Keywords:** Climate change, Global warming, Mortality, Urban heat islands, Bibliometric analysis, Environmental risks

## Abstract

**Background:**

With the increasing impacts of climate change, heatwaves are placing an enormous burden on health and social systems and threatening ecological diversity around the world. Heatwaves are increasing not only in frequency but also in severity and magnitude. They are causing the deaths of thousands of people. Research is needed on a multidisciplinary, supra-regional, and regional level.

**Methods:**

A detailed evaluation of the research conducted is not yet available. Therefore, this study provides a detailed insight into the publication landscape to identify key players, incentives, and requirements for future scientific efforts that are useful not only for scientists but also to stakeholders and project funders.

**Results:**

The number of publications on heatwaves is increasing, outpacing the trend of research indexed by the Science Citation Index Expanded. However, funding is lagging behind comparatively. Looking at absolute numbers, the USA, Australia, China, and some European countries have been identified as major players in heatwave research. If socio-economic numbers are included, Switzerland and Portugal lead the way. Australia and the UK dominate if the change in heatwave-exposed people is included. Nevertheless, exposure and economic strength of publishing countries were identified as the main drivers of national research interests. Previous heatwaves, in particular, have driven research efforts primarily at the national level.

**Conclusion:**

For an efficient monitoring or early detection system that also includes the economically weak regions, internationally networked efforts are necessary to enable preventive measures and damage limitation against heatwaves. Regardless of previous regional extreme heat events, research approaches should be focused to the global level.

## Background

Unusually hot weather events lasting several days are often referred to as heatwaves. Currently, they are one of the most dangerous environmental threats and can have enormous effects on all aspects of life, including individual and public health, food security, and the economy [1]. The observed effects of global warming, with climate change being the main driver, are also affecting extreme heat events, which are increasing in frequency, magnitude, duration, and measured temperatures. Whereby most land regions will certainly be affected in the future [2, 3]. Without human influence on climate change, some recent heat events most likely would not have occurred. It is estimated that heatwaves at least doubled due to human influence [4] and currently occur twice per decade. Until the early 2000s, they occurred only twice per century [5]. Marine heatwaves have also occurred twice as frequently since the 1980s [3]. The resulting impacts pose one of the most serious global health challenges of the 21st century and put many people at risk. In the decade prior to 2017, more than 166,000 people died worldwide as a result of extreme heat [1]. A study found that 37% of heat-related deaths are due to anthropogenic climate change [6]. From 2000 to 2016, about 125 million people more have been affected by heatwaves [1]. Low-income countries in particular, which lack response, adaptation, or preparedness capacity, face the most severe health risks. Studies of past heatwaves have shown their harmful effect on human health. Heatwaves, in the USA in 1995 [7], in Europe in 2003 [8], in South-East Australia in 2009 [9], in the Russian Federation in 2010 [10], in the United Kingdom (UK) in 2019, and in Canada in 2021 [11] resulted in the deaths of many thousands of people. The extreme heatwave of 2003, for example, claimed 14,800 lives over nine extremely hot days in France alone, 20 times more than the 1995 Chicago heatwave [12]. For regions already exposed to extreme heat, it must be assumed that a further increase in temperature will lead to conditions that are no longer viable for humans [13]. The urban heat island effect contributes to the predicted accumulations of heatwaves, especially in cities with growing populations, mainly affecting developing countries [14, 15].

Although the relationship between heat and health is difficult to determine due to the multitude of influences [16], some risk factors have been identified. For example, pre-existing conditions such as cardiovascular or respiratory disease increase the risk of the harmful effects of extreme heat [17]. Older people are also more at risk [18]. Certain working conditions additionally contribute to higher vulnerability to heat events, such as heavy physical labor or working outdoors. Social status may also contribute to increased vulnerability, such as poor housing or homelessness [19, 20]. Crop failures caused by heatwaves will certainly have an impact on societies worldwide [21].

It is important to consider that an increase in heat events does not only have an impact on humans. For example, it has led to mass invertebrate mortality in the past, and thus may affect biodiversity in the future [22].

Given the threat of even higher temperatures predicted in the coming years, the development and application of adaptation and preparedness measures are urgently needed. However, the current data situation does not allow a concrete assessment of the required measures and the priority target groups [23]. This requires a sound scientific basis that reaches all regions worldwide. An in-depth analysis of existing research and an assessment of scientific needs to address the threats of even more intense heatwaves to populations in all parts of the world is needed.

Therefore, this study aims to provide a comprehensive insight into the development and status quo of research activities worldwide. It is intended to give the necessary background for estimating the urgent scientific effort required, taking into account both the regions and populations most at risk and the multidisciplinary scope of the research required. The results will help scientists, project managers, stakeholders, and funders to plan constructive approaches focused on far-sighted solutions to address future heatwaves.

## Methods

### Methodological platform and data source

This study was conducted within the established bibliometric platform *New Quality and Quantity Indices in Science* (NewQIS) [24], which was developed to analyze important scientific topics in terms of their publication patterns. Its methodology combines proven approaches with newly developed topic-related parameters. Permanent further development ensures the validity of results discussed [25]. The integration of *Density Equalizing Map Projection* (DEMP) [26] of geographical results, allows for elaborate and fast information retrieval.

### Search procedure and data base generation

The WoS was searched for different synonyms or word variations for heatwaves: heatwave* OR “heat wave*” OR “extreme heat” OR “heat wave*” OR “heat disaster*.“ To reduce false-positive entries, the search was limited to the title of the publications. Here, the asterisks represent different word endings, and the Boolean operator combines the different terms to search for each of them. There was no time limit, and all types of documents were considered.

Additional search terms were used to further evaluate the retrieved articles. For example, to identify anthropogenic mentions in the history of heat wave research, the terms: anthropog* OR “man-made” OR “man-induced” OR “human-caused” were added as topic searches and sorted by date.

### Analyses and visualization of results

The metadata of all publications included in the database were analyzed using bibliometric parameters such as temporal evolution, publication counts and citation indices. Geographic distribution was analyzed to identify key incentives, actors, and funders at the global level. The keywords used represent the research focus of research. In addition, the most frequently assigned research areas by WoS categories were identified and analyzed by time and country.

Data from the World Bank (population, gross domestic product [GDP]) [27] and from the United Nations (gross domestic expenditure for research and development [GERD] [28] were used for weighted analyses regarding socioeconomic characteristics of countries. Spearman correlation analyses were performed between the country GDP and GERD as well as the population size and the number of researchers to validate the application of the socio-economic parameters.

To examine the burden of vulnerable population (> 65 years) to heatwaves in each country, a previous study evaluated data from a global and cross-national analysis. For this purpose, J. Chambers calculated the average change in heatwave days per person-day from 2010 to 2018 compared with a baseline period from 1986 to 2005 selected according to the approaches of the Intergovernmental Panel on Climate Change (IPCC) [29, 30]. In the present study, these data are related to the number of articles per country. Spearman correlation analysis was performed for the number of articles and the vulnerability of population thus defined.

By default, DEMPs are created in NewQIS studies. In this study, they were used to show the global patterns of heatwave research in terms of publication and citation counts, citation rate, and heat wave-related parameters. The results of the cluster analysis of keywords were visualized using the VOSviewer tool [31].

### Methodological limitations and strengths

Although the methodology used is based on established approaches [25], some limitations should be mentioned.

First, the analyses can only be as valid as the data source and the entries thus included allow. The characteristics of WoS as a data source do not allow for the inclusion of all relevant scientific papers, as only journals that meet the requirements of WoS are listed. However, the use of WoS has advantages over other data sources because only articles that have been qualitatively verified by the WoS requirements find their way into our study [32].

Furthermore, WoS provides citation counts that are important for the interpretation of the findings. The elaborated search term of bibliometric studies is generally a compromise that allows to find the majority of thematically related entries without including false entries that would destroy the representativeness of the results. As a result, a reduction in entries must be accepted.

Second, the already frequently discussed English bias of WoS must be mentioned as a limitation, since it favors English countries [25].

Third, the analyses on socioeconomic and heatwave-specific characteristics lack data for some countries because they are not provided by the data sources. To reduce this limitation, data sources that provided the best possible analysis were used.

## Results

A total of 3194 publications (n) on heatwaves from 1912 to 2021could be retrieved from the Web of Science Core Collection (WoS). The majority of them were published as articles (n = 2378, 74.45%). All other document types are only marginally represented.

### Chronological patterns

**Fig. 1:**
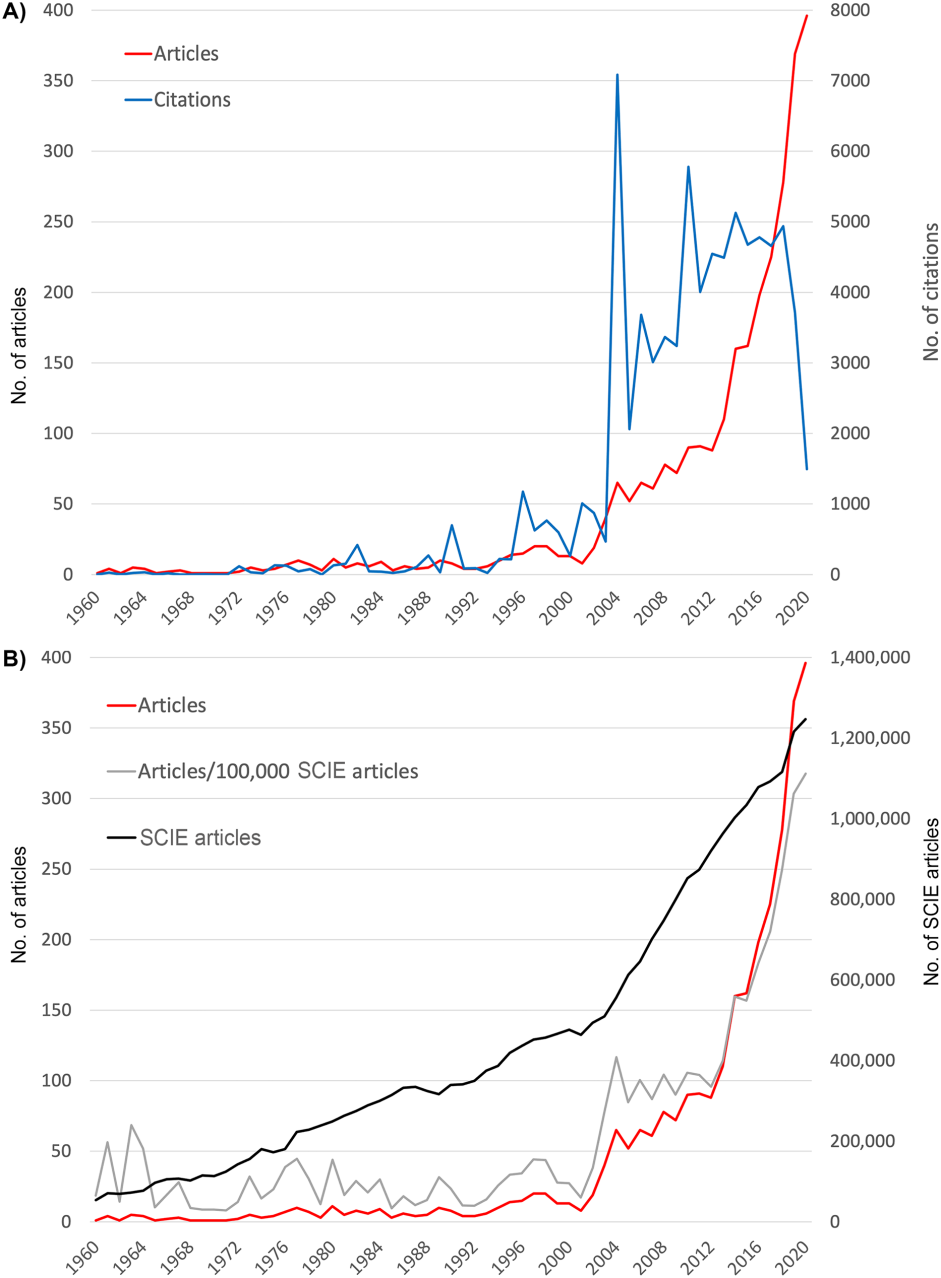
Development of publication numbers from 1960 to 2020. A) Number of publications and number of citations. B) Number of publications in relation to the number of articles included in the Science Citation Index Expanded (SCIE)

The first published article found in WoS dates from 1912 and deals with the occurrence of warm flows and the theory developed by H. von Picker to better predict such events [33]. Heatwaves were also treated only sporadically in the following years. Since the 1960s, a continuous number of publications could be observed, but at a very low annual level. Double-digit numbers per year were reached, with few exceptions, only in the 1990s. This was followed by significantly increasing publication numbers, reaching their maximum in 2020 with n = 396 articles on heatwaves (Fig. 1A). Compared to the development of all articles listed in the *Science Citation Index Expanded* (SCIE) of WoS, it is even more evident that the years 2003 and 2012 spurred global research efforts. (Fig. 1B).

Nevertheless, the impact of climate change was first mentioned in a 1997 article that discussed global warming projections in terms of predicting heat events such as the 1995 Chicago heatwave [34]. The possibility of anthropogenic influence was first raised in a risk analysis of the 2003 heatwave in France [35].

The number of citations (c) exploded in 2004, reaching a peak of c = 7086 (as of August 2021). Smaller annual peaks could also be identified in earlier years (1982: c = 418, 1990: c = 697, 1996: c = 1175). After 2004, years with high citation frequency could also be identified (2010: c = 5780, 2014: c = 5128). After 2018, in which c = 4936 citations was still reached, a clear decline in the annual citation figures could be demonstrated.

The year 2004 also stands out in heatwave research because it reached the highest annual citation rate (cr) of the entire evaluation period (cr = 109.01). Usually, these high citation rates are achieved in years with very low publication numbers, which is due to the quotient calculation and small numerator values. Only the year 2001 achieved a higher rate with cr = 125.87, but with only n = 8 publications.

However, the trend in publication effort across all SCIE-indexed research areas is also steadily increasing. The ratio of the number of heatwave articles to the number of SCIE articles shows values that, with few exceptions, remained consistently below a level of 50 publications per 100,000 SCIE publications until 2003. Beginning in 2012, the annual increase in heatwave publications exceeded that of SCIE-indexed research overall, as shown by the steep grey curve in Fig. 1B.

In addition, the three most cited articles were published in 2004. All were related to the 2003 European heatwave and were published in either Nature or Science. It is noteworthy that the other articles in the top 10 were published later, with one exception from 1996. This article dealt with the 1995 Chicago heatwave (Table 1).

**Table 1:** Most frequently cited publications, c = number of citations, NEJM = New England Journal of Medicine, NCAR = National Center for Atmospheric Research, ETH = Swiss Federal Institute of Technology Zurich, CDC = Center for Disease Control and Prevention, UKRI = UK Research and Innovation, EPA = Environmental Protection Agency, NIH = National Institutes of Health, EU = European Union, ANR = French National Research Agency

Authors	Year	c	Title	Journal	Institution	Funding Agency
Meehl, G.A., Tebaldi, C. (USA)	2004	2182	More intense, more frequent, and longer lasting heat waves in the 21st century	Science	NCAR	
Schar, C. et al. (Switzerland)	2004	1874	The role of increasing temperature variability in European summer heatwaves	Nature	ETH	
Stott, P.A., Stone, D.A., Allen, M.R. (UK)	2004	946	Human contribution to the European heatwave of 2003	Nature	University Reading	UKRI (UK)
Semenza, J.C. et al. (USA)	1996	774	Heat-related deaths during the July 1995 heat wave in Chicago	NEJM	CDC	
Anderson, B.G., Bell, M.L. (USA)	2009	719	Weather-Related Mortality How Heat, Cold, and Heat Waves Affect Mortality in the United States	Epidemiology	Yale University	EPA, NIH (USA)
Johnk, K.D. et al. (Netherlands, UK, Germany)	2008	590	Summer heatwaves promote blooms of harmful cyanobacteria	Global Change Biology	University Amsterdam	UKRI (UK)
Luber, G., McGeehin, M. (USA)	2008	546	Climate Change and Extreme Heat Events	American Journal of Preventive Medicine	CDC	
Fischer, E.M., Schar, C. (Switzerland, USA)	2010	534	Consistent geographical patterns of changes in high-impact European heatwaves	Nature Geoscience	ETH	EU
Garrabou, J. et al. (Spain, France, Italy)	2009	517	Mass mortality in Northwestern Mediterranean rocky benthic communities: effects of the 2003 heat wave	Global Change Biology	Spanish National Research Council	Ministry of Science (Spain), ANR (France)
Fischer, E.M. et al. (Switzerland, UK)	2007	508	Soil moisture - Atmosphere interactions during the 2003 European summer heat wave	Journal of Climate	ETH	UKRI (UK)

### Research foci

**Fig. 2:**
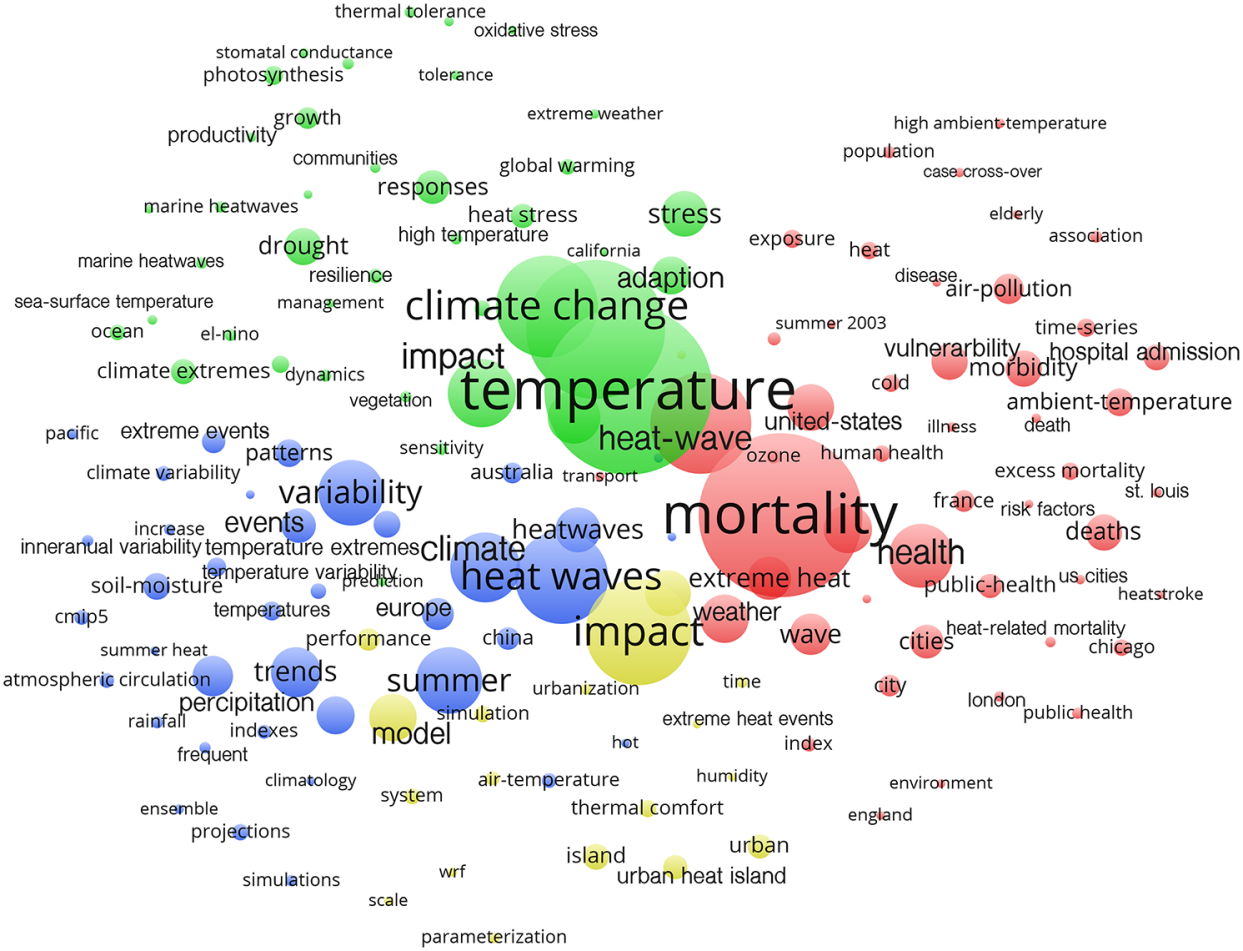
Cluster analysis of the keywords used (threshold: at least 20 occurrences). Thematical affiliations: red cluster – impact on human health and risk factors, blue cluster – high atmospheric temperatures and climate, green cluster – high oceanic temperatures, and impact on vegetation, yellow cluster –urbanization and habitation, modelling

The analysis of the keywords used in the publications on heatwaves allowed the interpretation of research foci. Four clusters with different thematic references could be identified (Fig. 2). These clusters also refer to different regions, which are mainly used as keywords. For example, the human impacts cluster (red) has research foci in the USA and the UK, while the atmospheric effects cluster (blue) can be associated with the regions of Europe, Australia, and China. The cluster of oceanic warming and the effects on flora (green) is in particular associated with California (USA) and South America (“El-Niño”). The cluster relating to urban heat islands (yellow) has no specific regional link.

**Fig. 3:**
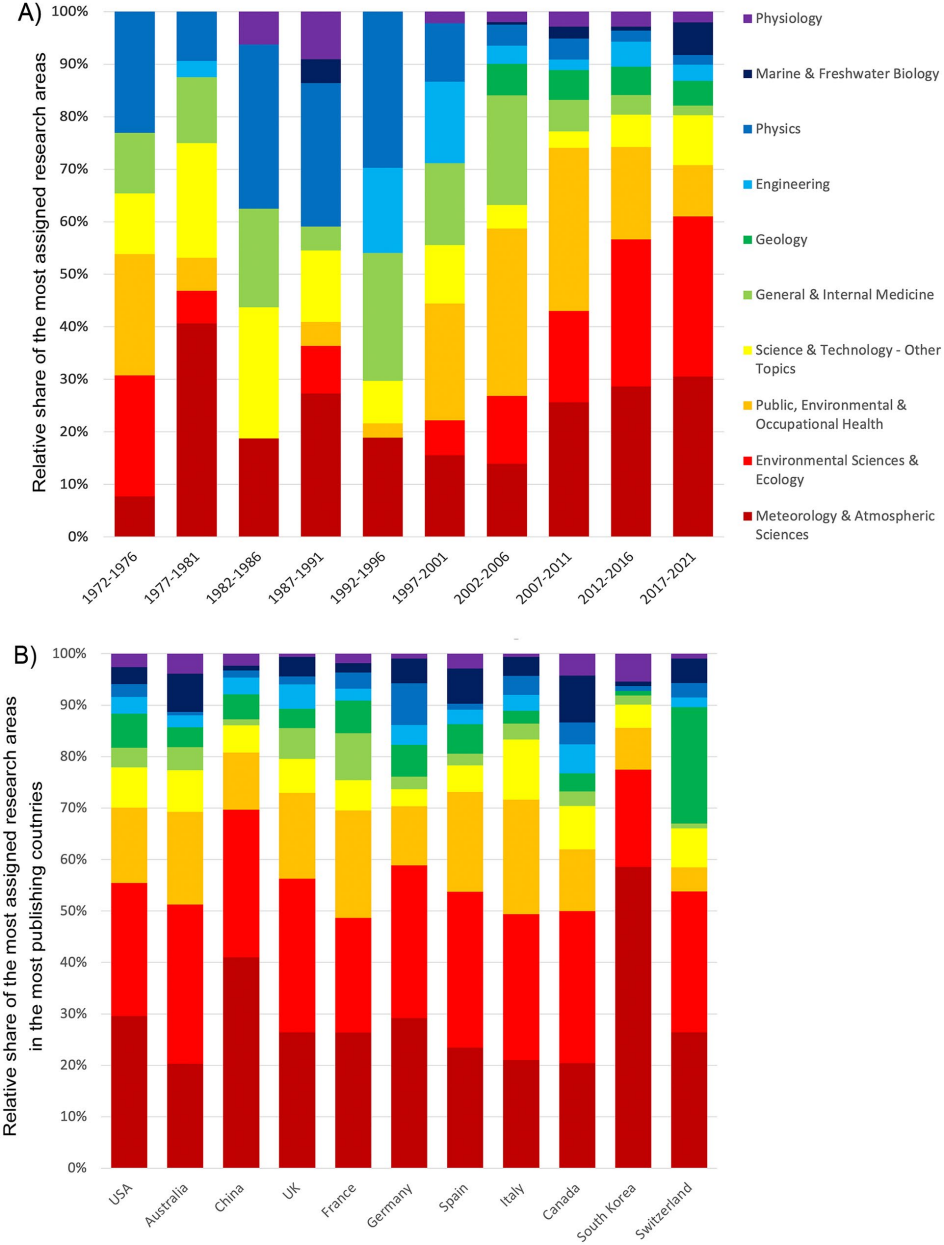
Analysis of research areas (categories of WoS). A) Relative distribution of the most assigned research areas in 4-year intervals from 1972 to 2021. B) Relative distribution of the most assigned research areas in the articles of the most publishing countries

Evaluation of the most frequently assigned research areas revealed “Meteorology & Atmospheric Sciences” as the most frequently covered area with n = 846 publications, followed by “Environmental Science & Ecology” (n = 781), “Public, Environmental & Occupational Medicine” (n = 468), “Science & Technology - Other Topics” (n = 247), and “General & Internal Medicine” (n = 155). Figure 3 shows the evaluation of the occurrence of research areas in heatwave research according to their development over time (Fig. 3A) and their distribution in the publications of the countries with the strongest publications (Fig. 3B).

### Geographical patterns

Of the total number of publications on heatwaves, n = 2930 articles (91.73%) could be assigned to a country of origin and were thus included in the geographical analyses of 96 publishing countries.

**Fig. 4:**
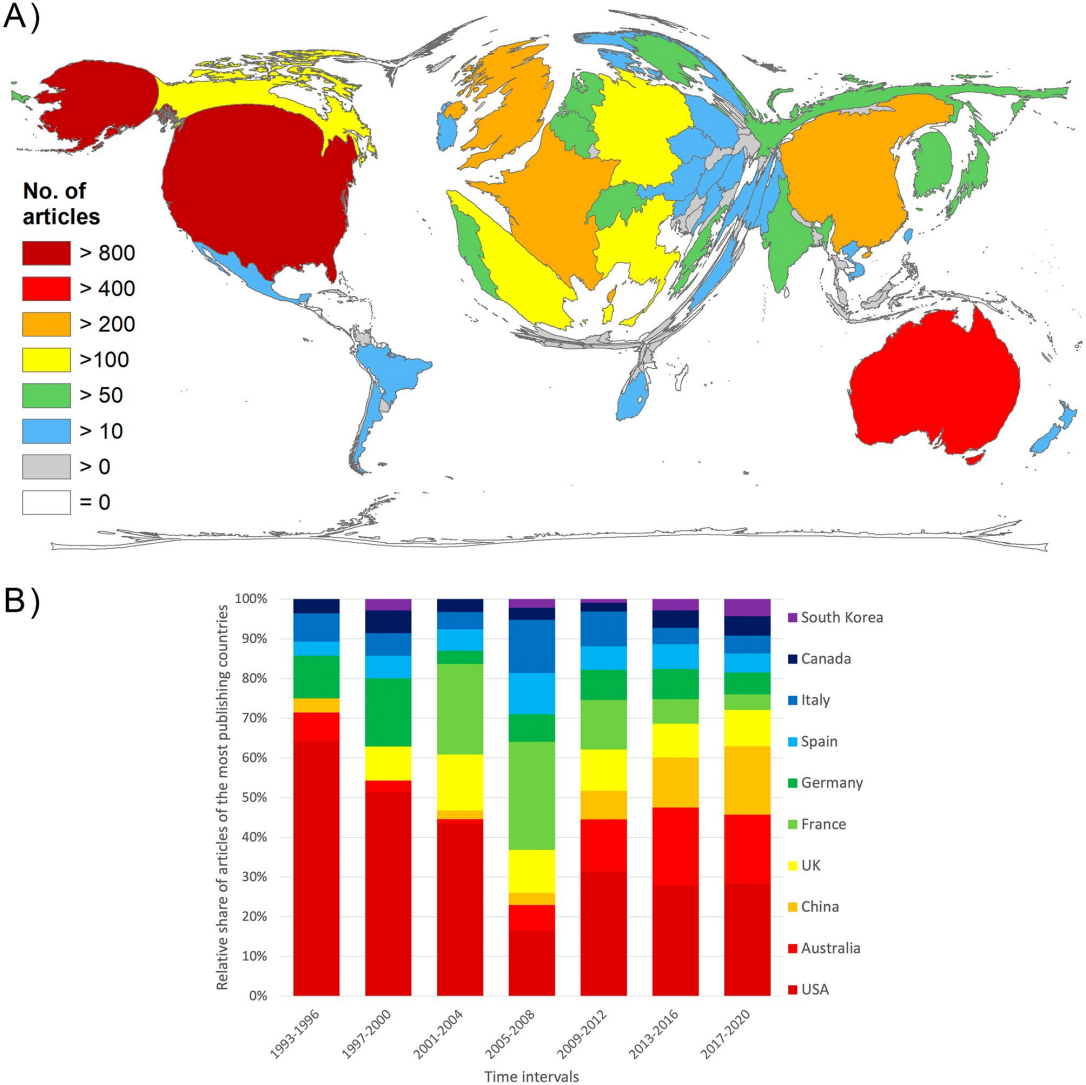
Countries’ publication performance on heatwaves. A) Number of articles per country. B) Development of the share of articles by the ten most publishing countries in 4-year intervals from 1993 to 2020

The results of the overall global publication performance on heatwaves show a clear predominance of the USA as the country with the highest number of publications (n = 886), followed by Australia (n = 455), China (n = 389), and some European countries (UK n = 289, France n = 239, Germany n = 200). The DEMP (Fig. 4A) indicates the bias of the world map towards these regions. A look at the development over time reveals the decrease in the US-American and the increase in the Australian and the Chinese shares of the global heatwave publications. The high proportion of French articles in the first decade of the 2000s is striking. In particular, the period from 2005 to 2008 is notable for France being the most publishing country during this period, with Italy following shortly after the USA in third place (Fig. 4B).

**Fig. 5:**
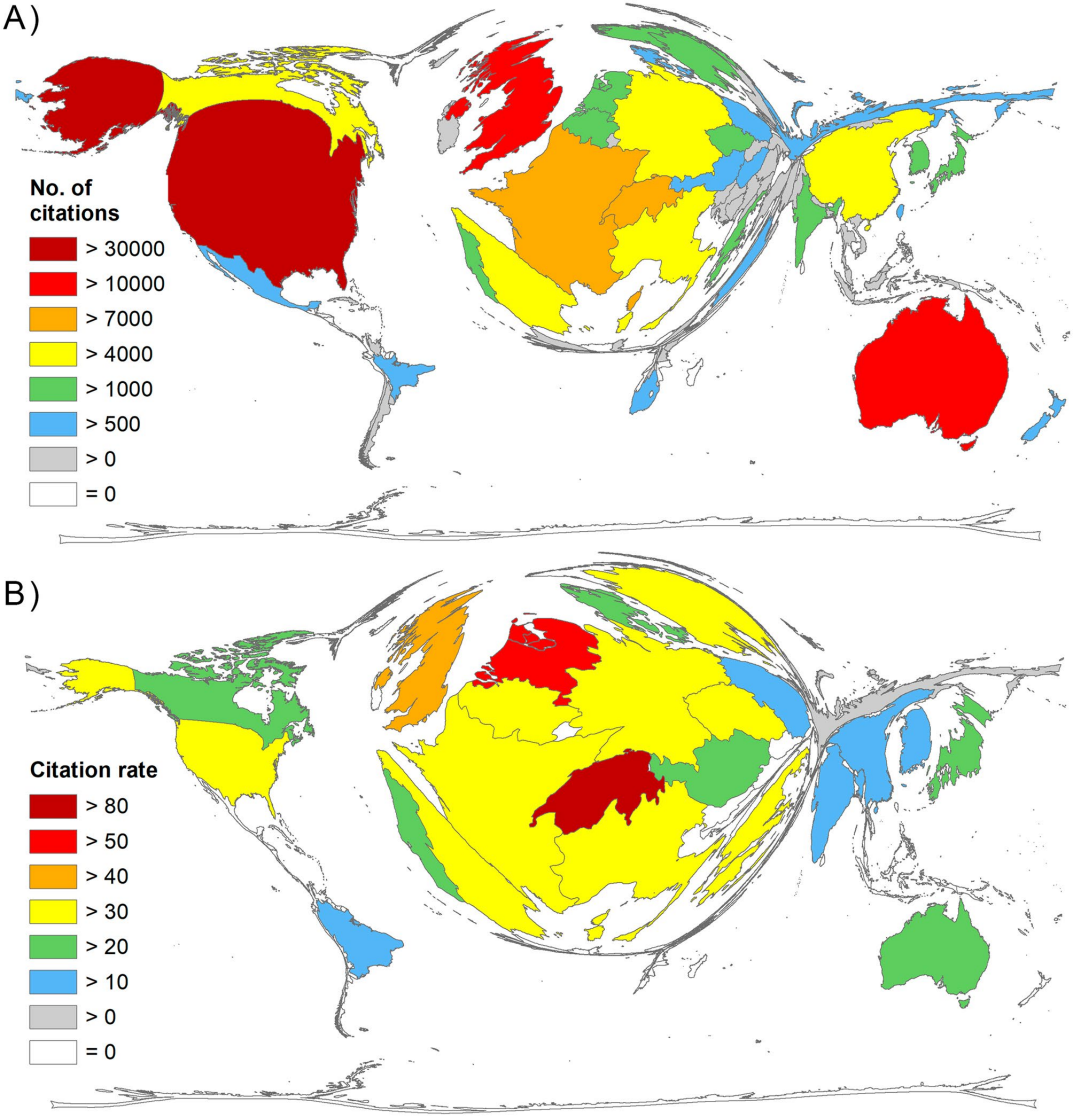
Citation parameters. A) Number of citations. B) Citation rate (number of citations / number of articles), threshold: 30 articles on heatwaves

The number of citations per country corresponds to the publication numbers and shows the dominance of the USA (c = 31,986), Australia (c = 13,079), and Europe with the UK leading with c = 11,919. France reached c = 9073, and Switzerland – in 5th place – c = 8078 (Fig. 5A), justifying the first rank of the Swiss publications in terms of the citation rate (cr = 85.94) when applying the methodological threshold of at least 30 publications on heatwaves per county. The Netherlands achieved the second highest citation rate (cr = 51.63), followed by the UK (cr = 41.24), Italy (cr = 39.52), and Belgium (cr = 38.38) to name the top 5 (Fig. 5B).

### Inclusion of national socio-economic features

**Fig. 6:**
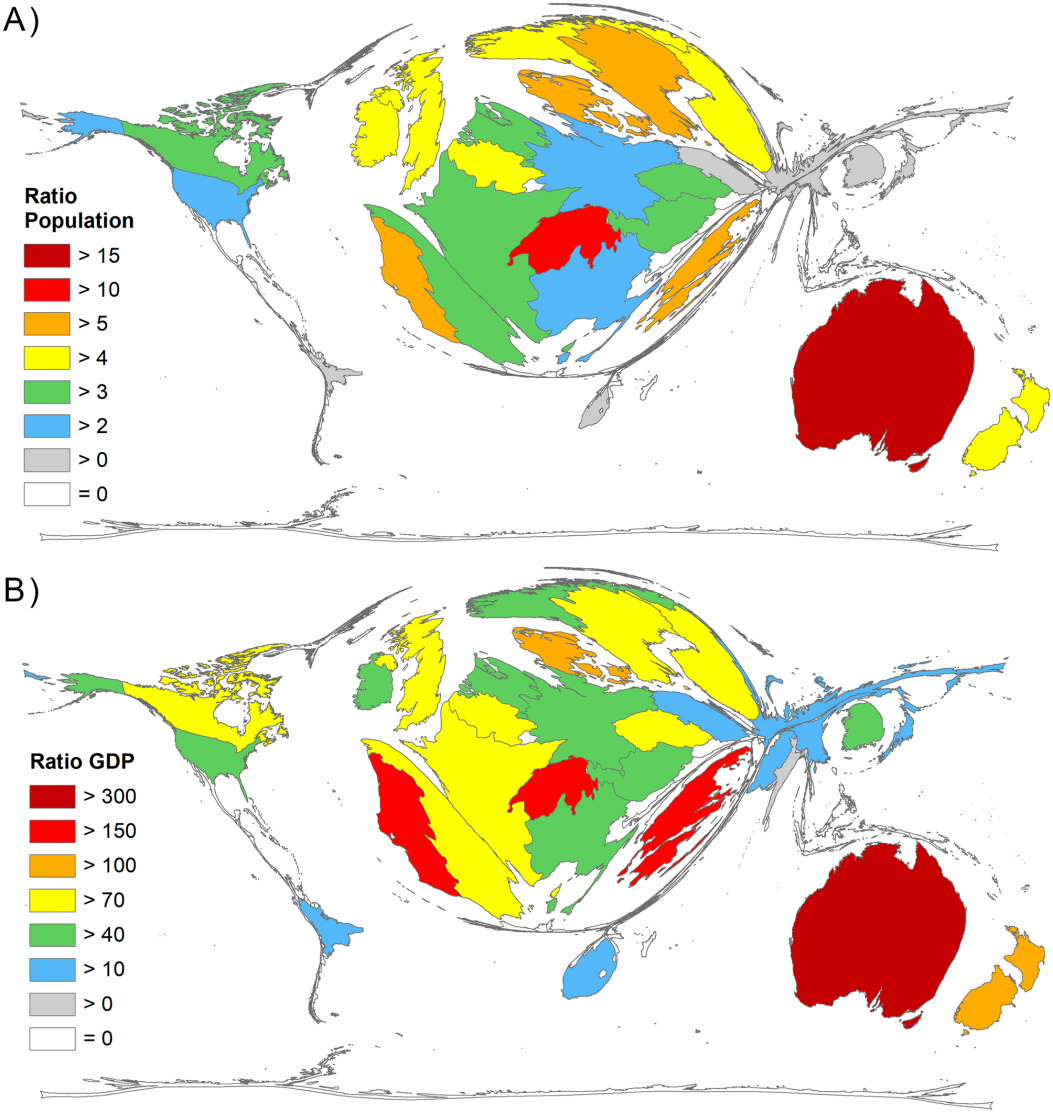
Socio-economic parameters, threshold: 30 articles. A) Ratio Population (R_POP_): Number of articles / population size in mill. B) Ratio GDP (RGDP): Number of articles / Gross Domestic Product (GDP) in 1000 bn US-Dollars [28]

To include the demographic and economic characteristics of the countries with a publication volume of more than 20 publications on heatwaves (threshold) in the evaluation, the number of articles was set in relation to the countries’ population size (R_POP_ = Number of articles / total population in mill.) and the gross domestic product (R_GDP_ = Number of articles / GDP in 1000 bn US-Dollars). This presents a different picture of the global research landscape. For the two socioeconomic parameters, Australia is at the top (R_POP_ = 18.05, R_GDP_ = 336.43). For the number of publications per million inhabitants (R_POP_), the ranking continues with Switzerland (R_POP_ = 10.94), Portugal (R_POP_ = 6.16), Denmark (R_POP_ = 6.06), and Sweden (R_POP_ = 5.28) (Fig. 6A). In terms of publications per GDP (R_GDP_), Portugal (R_GDP_ = 168.21) ranked second, followed by Greece (R_GDP_ = 163.45), Switzerland (R_GDP_ = 154.42), and New Zealand (R_GDP_ = 106.42) (Fig. 6B).

Table 2 provides an overview of the ranking of socio-economic ratios. The values of the corresponding data such as GDP for research and development (GERD) and number of researchers are highly significantly correlated with country GDP (Spearman r = 0.90, p < 0.0001) and population size (Spearman r = 0.62, p < 0.0001), resulting in almost equal rankings.

**Table 2:** Ranking of socio-economic ratios R_POP_ (number of articles/population in mill.) and R_GDP_ (number of articles/GDP in 1000 bn US-Dollars), threshold ≥ 20 articles on heatwaves, sorted by R_POP_., ppp = purchasing power parity

Country	Articles	Population in mill	GDP 1000 bn PPP	R_POP_	Rank_POP_	R_GDP_	Rank_GDP_
Australia	455	25.203	1.35	18.05	HI 1	336.43	HI 1
Switzerland	94	8.591	0.61	10.94	HI 2	154.42	HI 4
Portugal	63	10.226	0.37	6.16	HI 3	168.21	HI 2
Denmark	35	5.772	0.35	6.06	HI 4	100.54	HI 6
Sweden	53	10.036	0.57	5.28	HI 5	92.32	HI 7
Greece	55	10.473	0.34	5.25	HI 6	163.45	HI 3
New Zealand	23	4.783	0.22	4.81	HI 7	106.42	HI 5
Norway	25	5.379	0.36	4.65	HI 8	69.95	HI 15
Belgium	52	11.539	0.63	4.51	HI 9	83.01	HI 10
UK	289	67.53	3.26	4.28	HI 10	88.77	HI 8
Finland	23	5.532	0.28	4.16	HI 11	81.18	HI 11
Ireland	20	4.819	0.44	4.15	HI 12	45.87	HI 19
Canada	138	37.411	1.93	3.69	HI 13	71.51	HI 14
Austria	33	8.955	0.52	3.69	HI 14	62.89	HI 16
Spain	172	46.737	1.99	3.68	HI 15	86.55	HI 9
France	239	64.991	3.32	3.68	HI 16	72.09	HI 13
Netherlands	62	17.097	1.03	3.63	HI 17	59.93	HI 18
Czech Republic	34	10.689	0.45	3.18	HI 18	74.85	HI 12
USA	886	328.239	21.37	2.70	HI 19	41.45	HI 22
Italy	162	60.55	2.66	2.68	HI 20	60.79	HI 17
Germany	200	83.517	4.66	2.39	HI 21	42.92	HI 21
South Korea	97	51.225	2.22	1.89	HI 22	43.60	HI 20
Poland	41	37.888	1.30	1.08	HI 23	31.56	HI 23
Russia	77	145.872	4.28	0.53	UMI 1	17.98	UMI 2
Japan	58	126.246	5.46	0.46	HI 24	10.62	HI 24
South Africa	26	58.558	0.76	0.44	UMI 2	34.16	UMI 1
China	389	1433.784	23.46	0.27	UMI 3	16.58	UMI 3
Brazil	48	211.05	3.22	0.23	UMI 4	14.91	UMI 4
Pakistan	23	216.565	1.06	0.11	LMI 1	21.74	LMI 1
India	73	1366.418	9.61	0.05	LMI 2	7.59	LMI 2

### Inclusion of data on vulnerability to heatwaves

**Fig. 7:**
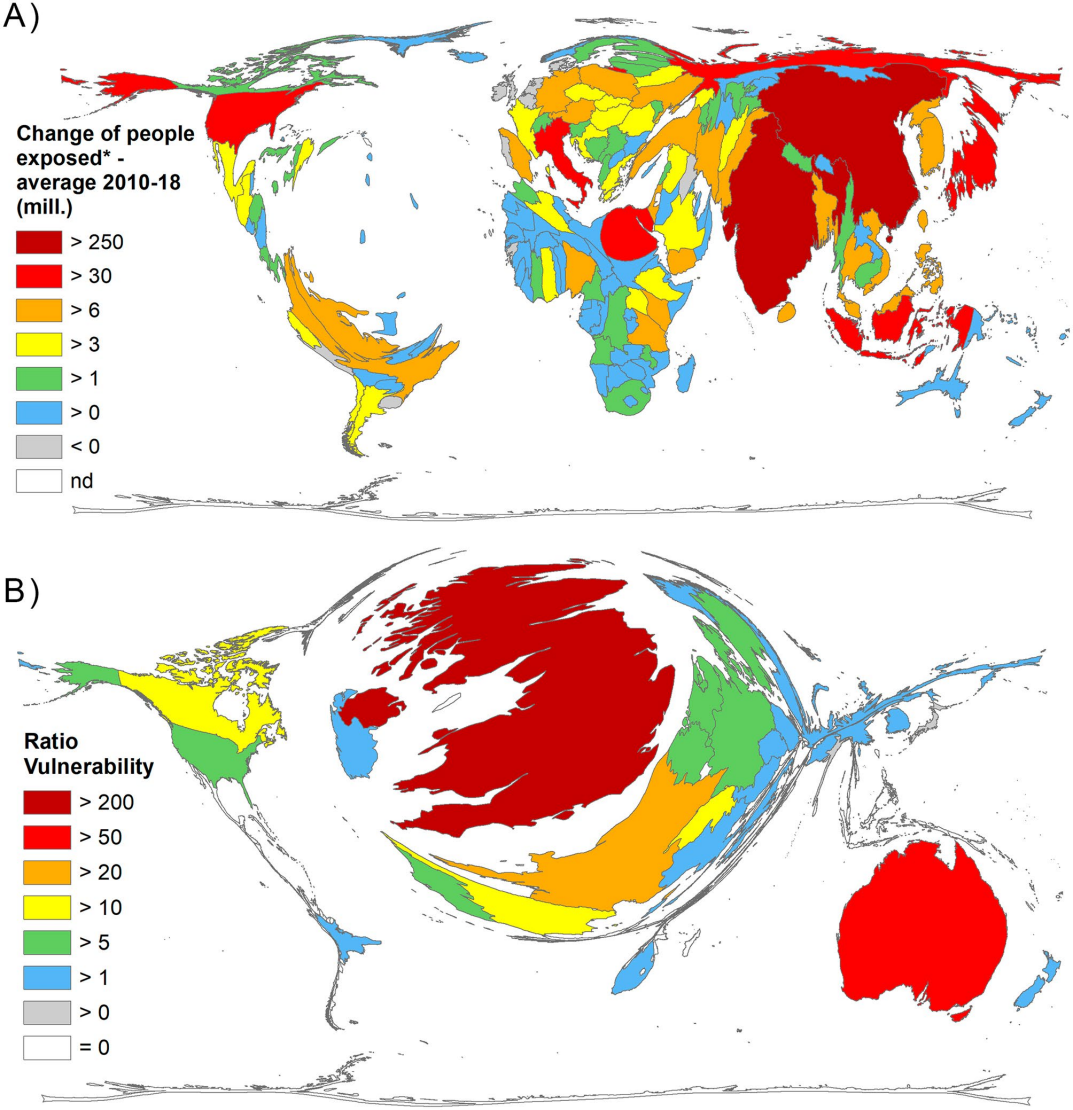
People exposed to heatwaves. A) Average change of people exposed, * from 2010 to 2018 in million persons per day (29). B) Ratio of number of articles to the average change (threshold: 20 articles) [29]

Due to the large regional differences in terms of vulnerability to heatwave events, we included heatwave-related parameters in the analysis. For this purpose, the change in the number of people exposed to heatwaves on average from 2010 to 2018 was the basis [29]. A look at Chambers’ figures shows that the exposures of the populations in China, India, Japan, the USA, Indonesia, Russia, Egypt and Italy are rising sharply (Fig. 7A). If the number of articles (threshold: at least 20 articles on heatwaves per country) is related to these figures, the ranking changes considerably (Fig. 7B). Now, the UK distinctly dominates the landscape (R_vuln_ = 222.42) as the average change in exposed population here decreased over the time frame. Australia (R_vuln_ = 57.19) is second, followed by France (R_vuln_ = 23.18), Canada (R_vuln_ = 52), and Switzerland (R_vuln_ = 11.64).

**Fig. 8:**
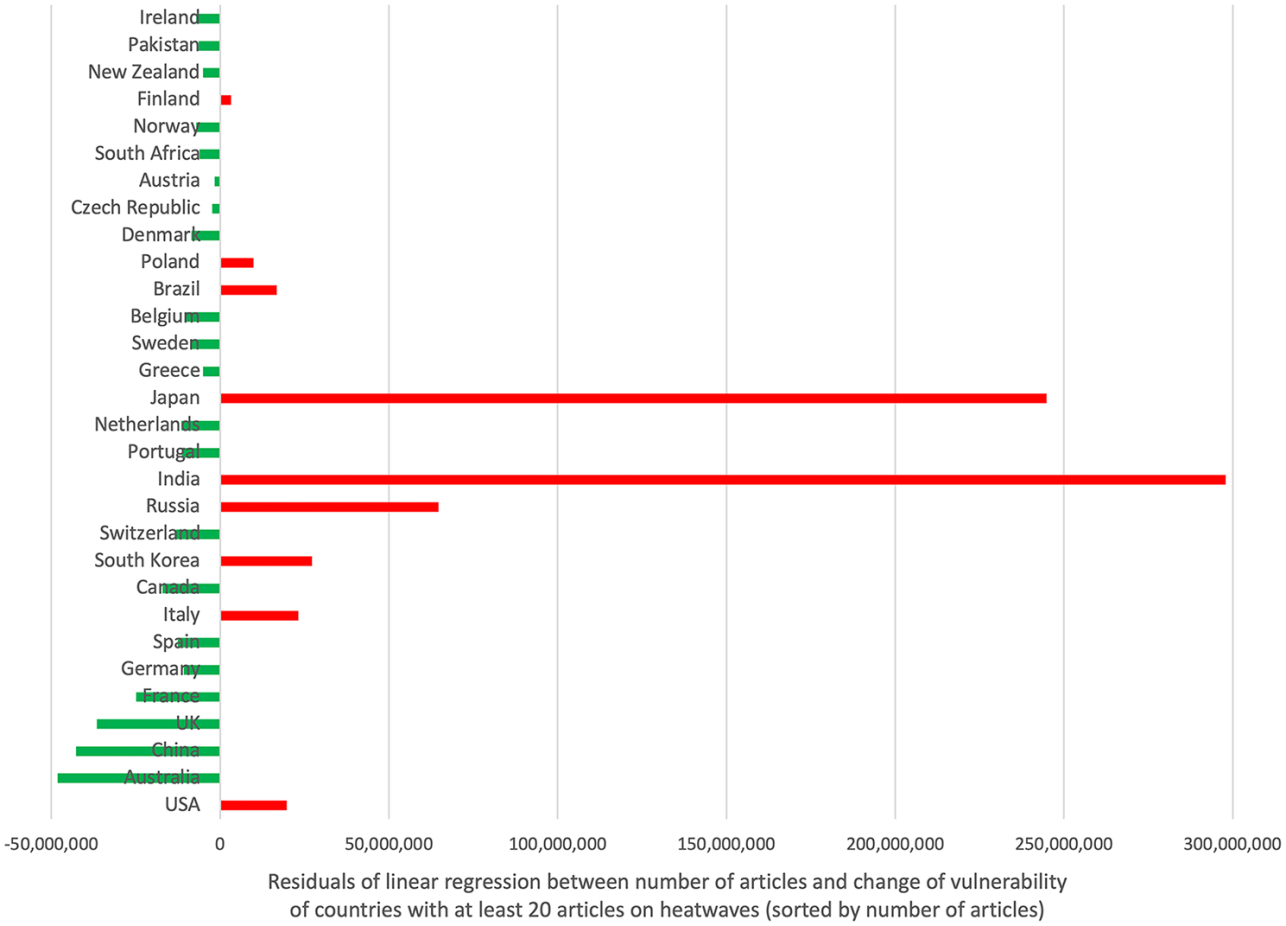
Residuals of linear regression between the number of articles and the vulnerability [29]

Table 3 summarizes the ranking of R_vuln_ of countries with at least 20 articles on heatwaves (threshold). The correlation between the number of items and the change in vulnerability to heatwaves is significant (Spearman r = 0.44, p < 0.0001). The plot of the residuals shows the highest negative deviation (in favor of the number of articles: positive balance) for Australia, China, the UK, and France, while the highest positive deviations (negative balance) were detected for India, Japan, Russia, and South Korea (Fig. 8). The USA is also in the positive deviation range and thus has a rather negative balance.

**Table 3:** Ranking of R_vuln_ for countries with at least 20 articles on heatwaves (quotient of the number of articles and the number of people exposed to heatwaves on average from 2010–2018 per 100,000 people, for calculation reasons the denominator was summed with the mean value) [29]

Country	Articles	Change (avg. 2010-18)	(Avg + mean)/ 100,000	R_vuln_
UK	289	-5,699,393	1.30	222.42
Australia	455	957,339	7.96	57.19
France	239	3,311,564	10.31	23.18
Canada	138	1,355,027	8.35	16.52
Switzerland	94	1,077,751	8.08	11.64
Spain	172	8,617,450	15.62	11.01
Germany	200	13,178,646	20.18	9.91
USA	886	84,823,329	91.82	9.65
Netherlands	62	-377,043	6.62	9.36
Portugal	63	-243,176	6.76	9.33
Belgium	52	-441,244	6.56	7.93
Sweden	53	1,476,880	8.48	6.25
Denmark	35	-60,623	6.94	5.04
Greece	55	5,280,288	12.28	4.48
Norway	25	435,897	7.43	3.36
Italy	162	41,666,070	48.66	3.33
South Africa	26	1,496,577	8.50	3.06
Ireland	20	-148,962	6.85	2.92
New Zealand	23	977,445	7.98	2.88
Czech Republic	34	5,973,167	12.97	2.62
Finland	23	2,203,416	9.20	2.50
Austria	33	6,502,812	13.50	2.44
South Korea	97	35,000,692	42.00	2.31
Poland	41	17,685,757	24.68	1.66
Brazil	48	25,043,218	32.04	1.50
Pakistan	23	9,201,671	16.20	1.42
China	389	310,076,822	317.08	1.23
Russia	77	66,011,200	73.01	1.05
Japan	58	110,389,844	117.39	0.49
India	73	255,503,719	262.50	0.28

Fig. 8. Residuals of linear regression between the number of articles and the vulnerability [29]

### Funding of heatwave research

Of all n = 3194 articles, n = 2905 articles (90.95%) received financial support, with 4439 grants (g) awarded. In total, 54 governments supported heatwave research with g = 3170 grants, representing 71.41% of all grants awarded, including provincial and municipal funds (Table 4). The USA supported the most studies with g = 747 (0.84 grants per article), followed by China (g = 680, 1.75 grants per article). The Australian government made a financial contribution of g = 416 articles, the UK government funded g = 213 articles, and the Spain government funded g = 118 articles (Table 4).

**Table 4:** Governmental grants

Country	Grants	Articles	Grants/articles
USA	747	886	0.84
China	680	389	1.75
Australia	416	455	0.91
UK	213	289	0.74
Spain	118	172	0.69
South Korea	108	97	1.11
Canada	96	138	0.70
France	89	239	0.37
Germany	76	200	0.38
Brazil	62	48	1.29
Portugal	59	63	0.94
Japan	47	58	0.81
Italy	39	162	0.24
Belgium	39	52	0.75
Czech Republic	34	34	1.00
India	32	73	0.44
Sweden	32	53	0.60
Russia	29	77	0.38
Switzerland	21	94	0.22
Netherlands	20	62	0.32
Poland	13	41	0.32
Austria	13	33	0.39
Denmark	9	35	0.26
Greece	5	55	0.09

The correlation (Spearman) between the number of articles and the number of grants per publishing country is highly significant (r = 0.86, p < 0.0001).

International or bilateral grants were awarded 404 times, with the European Commission (g = 287) being the most frequently funding international organization. The highest volume of funding from governmental organizations was provided by the USA with the National Science Foundation (NSF) with g = 224 and the National Institutes of Health (NIH) with g = 186 funds. China’s NSFC funded 220 times, the UK Research Institute (UKRI) funded 209 times, and the Australian Research Council (ARC) funded 171 times.

In addition to government funding, other organizations also fund heatwave research, although some are not completely independent of government support (Table 5). With more than 10 grants each, two Australian universities (University of Western Australia, University of Adelaide), two US universities (University of Michigan, University of California), and one Belgian university (KU Leuven) were the largest university contributors to heatwave research.

**Table 5:** Non-governmental grants

Non-Governmental	Number	Grants
Universities*	211	460
Foundations, Trusts	94	119
Companies	62	81
Societies	16	29
Research Institutes	21	25
Associations	6	23
Networks, Alliances	8	8
Banks	3	5
Hospitals	4	4
Crowdfunding	1	1

* Some universities are partly governmental funded

## Discussion

Until 2003, the relationship between the number of SCIE articles and heatwave-related articles was consistent. This is also true for 2004 to 2012, when the number of articles on heatwaves increased relatively stronger. The first sharp increase in publication numbers was due to the 2003 heatwave in Europe, which caused many deaths. This is also reflected in the relative increase in European publications in the evaluation interval for this time period and in subsequent years. In particular France and Italy, have increased their research on heatwaves after this heat summer, which was the hottest in Europe since 1500 [36]. The scientific efforts of European countries during this period were also due to the large number of associated mortalities in these countries, reaching 14,800 deaths in just nine days in France alone [12]. In total, about 70,000 people died as a result of the heatwave in 2003. In the European part of Russia, approximately 55,000 people fell victim to the heatwave of 2010 [37]. This, along with the South-East Australian heatwave of 2009, contributed to the citation peak in 2010. Especially when compared to the SCIE indexed publications, the sharp increase in articles on heatwaves in 2012 is exceptional and is certainly driven by the acceptance of the increasing frequency and devastating nature of heatwaves in the light of previous events. The UN body for the scientific assessment of climate change, the Intergovernmental Panel on Climate Change (IPCC), also confirmed in 2012 that the frequency of heatwaves is very likely to increase in most land areas [38].

Looking at the year-to-year trend in citation numbers, there are some peaks that can also be attributed to severe heatwaves. For example, the 1995 Chicago heatwave led to the 1996 citation peak, in part because this heatwave was often used as comparison for later heatwaves [12]. The overwhelmingly large 2004 peak is clearly related to the 2003 European heatwave, and the 2010 peak is certainly related to the 2009 and 2010 heatwaves in Australian and Russian, respectively. The dynamics of citation numbers are usually characterized by a sharp decline in citation counts about 7 to 8 years before the date of evaluation [25], as newer articles have not yet had time to accumulate maximum citation counts. This development is not observed in heatwave research. Here, the decline did not start earlier than 2018. Therefore, the peaks of 2014 and 2018 should also be considered exceptional and demonstrate a dynamic in the citation patterns of previous years, marking the high interest in this field of science due to the high rate of record-breaking heatwaves. In the last years of the period studied, China’s influence is clearly noticeable. China has not only published the second most articles in recent years, but also cites almost as many as the USA. Compared to the articles from USA or European, the Chinese publications are more concerned with regional heat events. The Australia-related articles, like the Chinese ones, were mostly written by national author groups. Nevertheless, the increased regional scientific interest is noticeable in every country with high publication volume on heatwaves.

It is not unusual that the countries with the most articles are the USA, Australia, China, and European countries. However, Australia’s high involvement is exceptional compared to other research areas [25] and is due to its extreme exposure to heatwaves. Russia’s exceptionally high ranking of 12th in the world is due to the extreme heatwave of 2010 and the mortality it caused. Yet this heatwave is more frequently discussed in US publications than in Russian ones.

A mismatch between the health effects of heatwaves and research effort has already been identified in a previous systematic review of 188 studies [15]. It shows the discrepancy between research in high-income and middle- or low-income countries. It also highlights research priorities in mid-latitude countries. A discrepancy could be identified between research efforts on heatwaves and morbidity and the population at risk. Here, tropical regions and some countries in Africa, South America, the Middle East, and Eastern Europe are underrepresented [15]. The results of the present study confirm these findings.

The association between a country’s publication output and its corresponding funding is not astonishing. Despite high exposure, low-economy countries do not participate as much as countries with high economic power. An analogy can be drawn between changes in the vulnerability of populations at risk, adverse health effects, and the scientific effort of the publishing countries. This is due, among others, to the high and increasing proportion of elderly people and the increasing number of people living in urban areas in high-income countries [29]. Great engagement in heatwave research without much change in the number of people at risk can be attributed to the UK and Australia. This can be explained by the stable ratios between the number of articles and a consistent negative or a very small increase in heatwave exposure of vulnerable populations in these countries.

If the economic strength of the publishing countries is taken into account, Australia is also in the lead, followed by Greece, Switzerland and Portugal. Along with the Scandinavian countries, these countries are also far ahead when population size is included. The influence of the Swiss working group at ETH Zurich of the Department of Environmental Systems Science with scientists C. Schär and E.M. Fischer, who participated in three of the ten most cited articles [39–41], explains Switzerland’s dominant rank in citation rates. Portugal and Greece suffered extremely from wildfires associated with the 2003 heatwave, with an additional 60% of agricultural land burned in Portugal. The European wildfires caused financial damage of more than 13 billion euros, one billion euro in Portugal alone [42, 43].

The impact on the Mediterranean region was also the focus of the much-cited study by Garrabou et al. [22] of Spain, France, and Italy, who are major players in heatwave research. The UK participated in three of ten articles, as was Switzerland. One of this articles was co-authored with Germany and the Netherlands and addresses the heatwave-induced blooms of toxic cyanobacteria leading to mass mortality of fish and birds [4]. However, the USA also had the largest share of the most cited articles, five of ten, including the most cited 2004 article by Meehl et al. anticipating more frequent and intense heatwaves over North America and Europe [44]. That is consistent with recent events in Canada, where the temperatures soared above 49 °C and caused the deaths of hundreds of people. This underscores the danger of heatwaves, which will become even more hazardous in the future.

The evaluation of the research areas addressed shows that studies on atmospheric and environmentally relevant topics have recently increased, while other areas play more and more a minor role. In particular, the social sciences and economics fall extremely behind in comparison to other fields. This can also be seen in the relative distribution of research fields in the individual countries, where the strong fields also dominate. Only in Switzerland does “geology” take on a greater role as the third frequently addressed area.

It has been noted that there is a lack of observational data to conduct valid studies of heatwaves. Meteorological stations are often not available, so representative models cannot be constructed. It has also been noticed that there is too little research and too little funding in this currently small area of research [45]. Although this study identified a broad network of funding sources that correlates with the total number of publications, the comparison with research funding in other research areas is startling. In particular, the governments of European countries, which will certainly be more affected by heatwaves in the future, show a relatively low proportion of funding per article. It is imperative to raise awareness of this threat to provide an adequate basis for modelling, predictions, and thus preventing many deaths in the future. Scientific approaches need to be multidisciplinary and international in scope to enable global monitoring for close-meshed predictions and strategies to protect the countries’ populations.

## Conclusions

As heatwaves are certain to become more frequent and severe due to the climate change, the development of appropriate warning and protection measures is essential, especially for those countries with large vulnerable populations. That is more likely to be the case in more affluent regions, where the number of elderly people is steadily increasing. In addition, the increase in extreme heat events is particularly threatening to countries where temperatures are usually well below life-threatening levels. Here, lifestyles, air conditioning in private and public spaces, and working conditions and hours are not designed for high heat. In many warmer regions, on the other hand, living conditions are more adapted to life in high temperatures. Therefore, the vulnerability to the increase and intensity of heatwaves and the growing population at risk will affect populations around the world.

Based on the results of our study, it can be said that addressing this challenge will require coordination of multidisciplinary scientific efforts at the global level by all stakeholders, including government agencies and scientific institutions. Balanced research approaches require intergovernmental efforts and a broader range of funding, not just focused on local conditions.

## Data Availability

The bibliometric data is owned by and has been obtained from the Web of Science database. Therefore, authors are not allowed to share the data publicly or privately. Any researcher with access to the Web of Science database can obtain the data using the methods described in the paper.

## Abbreviations

ARC: Australian Research Council
DEMP: Density Equalizing Map Projections
GDP: Gross Domestic Product
GERD: Gross Expenditures for Research and Development
IPCC: Intergovernmental Panel on Climate Change
NewQIS: New Quality and Quantity Indices in Science
NIH: National Institutes of Health
NSF: National Science Foundation
NSFC: National Science Foundation of China
UKRI: United Kingdom Research Institute
WoS: Web of Science
